# Elastic Mechanical Properties of 45S5-Based Bioactive Glass–Ceramic Scaffolds †

**DOI:** 10.3390/ma12193244

**Published:** 2019-10-04

**Authors:** Francesco Baino, Elisa Fiume

**Affiliations:** Institute of Materials Physics and Engineering, Applied Science and Technology Department, Politecnico di Torino, Corso Duca degli Abruzzi 24, 10129 Torino, Italy; elisa.fiume@polito.it

**Keywords:** bioactive glass, scaffold, bioceramics, mechanical properties, elastic modulus, porosity, Modelling

## Abstract

Porosity is recognized to play a key role in dictating the functional properties of bioactive scaffolds, especially the mechanical performance of the material. The mechanical suitability of brittle ceramic and glass scaffolds for bone tissue engineering applications is usually evaluated on the basis of the compressive strength alone, which is relatively easy to assess. This work aims to investigate the porosity dependence of the elastic properties of silicate scaffolds based on the 45S5 composition. Highly porous glass–ceramic foams were fabricated by the sponge replica method and their elastic modulus, shear modulus, and Poisson’s ratio were experimentally determined by the impulse excitation technique; furthermore, the failure strength was quantified by compressive tests. As the total fractional porosity increased from 0.52 to 0.86, the elastic and shear moduli decreased from 16.5 to 1.2 GPa and from 6.5 to 0.43 GPa, respectively; the compressive strength was also found to decrease from 3.4 to 0.58 MPa, whereas the Poisson’s ratio increased from 0.2692 to 0.3953. The porosity dependences of elastic modulus, shear modulus and compressive strength obeys power-law models, whereas the relationship between Poisson’s ratio and porosity can be described by a linear approximation. These relations can be useful to optimize the design and fabrication of porous biomaterials as well as to predict the mechanical properties of the scaffolds.

## 1. Introduction

Bioactive man-made materials show great promise in bone tissue engineering strategies as valuable and versatile options to replace transplant substances (autografts, allografts, and xenografts) for grafting [1]. These biomaterials are often produced 3D scaffolds, which act as templates to support and guide the regeneration of healthy tissue at the injured site [2]. Scaffolds for bone repair should fulfill a set of physico-chemical and biological properties, including the high biocompatibility and the release of non-toxic by-products during dissolution or degradation in vivo, the capability to bond to living bone through a stable interface, and the similarity to host bone in terms of pore-strut architecture and mechanical characteristics [3]. Assuming that an ideal scaffold should mimic the human healthy cancellous bone, a total porosity of 50 vol.% (at least) and a pore size above 100 µm are typically recommended to allow adequate perfusion of bio-fluids and nutrients, cell colonization, and vascularization [4]. 

Bioactive glasses are excellent candidates for making 3D scaffolds due to the fact of their versatility and exceptional capability of stimulating osteointegration and osteogenesis via the release of ionic dissolution products (primarily silicate and Ca^2+^ ions) that promote bone cell responses towards a path of regeneration and self-repair [5,6,7]. Convincing evidence is also emerging about the suitability of bioactive glasses in contact with soft tissues: in this regard, recent results in the field of wound healing proved the potential of bioactive glasses to promote skin repair and regeneration through the stimulation of angiogenesis [8,9]. 

However, producing strong bioactive glass scaffolds able to withstand the physiological loads in load-bearing sites is still a challenge. The first bioactive glass foams produced by Chen et al. [10] using 45S5 Bioglass^®^ powder as a starting material exhibited poor compressive strength (0.2–0.4 MPa) compared to the typical range of trabecular bone (2–12 MPa [11]). The main “technological” limitation of the famous 45S5 composition is that this glass has a narrow sintering window and tends to crystallize upon thermal treatment [12]. Therefore, the struts of 45S5 glass scaffolds are poorly densified [10] and, if the sintering temperature is increased, the crystalline phase resulting from partial devitrification decrease the material bioactivity [13]. The 45S5-based graded scaffolds having a highly porous outer shell surrounding a less-porous core were produced by combining sponge replication and polyethylene burn-off method in the attempt to increase the compressive strength (0.7 MPa) [14]. Wu et al. [15] fabricated stronger 45S5 Bioglass^®^ scaffolds by using rice husk as a sacrificial pore-forming agent (compressive strength in the range of 5 to 7 MPa), but the resulting porous structure was poorly interconnected and, thus, not very suitable for tissue engineering applications. Implementation of additive manufacturing methodologies, such as robocasting, led to a significant improvement of the compressive strength of 45S5 glass scaffolds (above 10 MPa [16,17]); however, these structures have a grid-like arrangement of channels that do not properly mimic the trabecular architecture of cancellous bone as glass scaffolds produced by foam replication successfully do.

Other bioactive glass compositions have been designed over the past two decades to overcome the limitations of 45S5 Bioglass^®^, which, however, remains the most commonly used biomedical glass for clinical use worldwide [18]. For example, bone-like glass or glass–ceramic foams based on CEL2 (43.8SiO_2_-23.6CaO-15.0Na_2_O-4.6MgO-6.1K_2_O-6.9P_2_O_5_ wt.%) [19], 13-93 (53SiO_2_-20CaO-6Na_2_O-5MgO-12K_2_O-4P_2_O_5_ wt.%) [20], and SCNA (57SiO_2_-34CaO-6Na_2_O-3Al_2_O_3_ mol.%) [21] glasses were shown to achieve a compressive strength of 5, 11, and 18 MPa, respectively.

Some studies have been focused on determining experimentally and modelling the relationship between compressive strength and porosity in bioactive glass and ceramic scaffolds [22,23,24]; on the contrary, other important mechanical properties, such the elastic modulus, have received relatively little attention. The elastic modulus is a key parameter for determining the biomechanical success of an implant as a good stiffness match between scaffold and host tissue allows favorable stress transfer along the interfacial zone, thus resulting in stable bonding and osteointegration [8]. The elastic modulus of 13-93 glass foams (total porosity 85 vol.%) produced by sponge replication was assessed to be around 3 GPa by Fu et al. [20]. In another study, Tagliabue et al. [25] reported that CEL2 scaffolds with total porosity of 65 and 85 vol.% exhibited an elastic modulus of 16 and 1.1 GPa, respectively. To the best of our knowledge, the elastic modulus of 45S5 Bioglass^®^ foams has not been determined in the literature so far. This is probably due to the presence of some technological problems, as the elastic modulus and the other elastic properties may be difficult to determine for brittle porous ceramics without a proper equipment, while the compressive strength is relatively easy to assess from the stress–strain curve. 

In the attempt to bridge this gap, the present work aims at determining the relationship between elastic properties—including the elastic modulus, shear modulus, and Poisson’s ratio—and total porosity of 45S5-derived glass–ceramic foams; modelling of experimental data is also presented and discussed. 

## 2. Materials and Methods

### 2.1. Fabrication of Glass-Derived Scaffolds

The 45S5 bioactive glass (45SiO_2_–24.5CaO–24.5Na_2_O–6P_2_O_5_ wt.%), used as a starting material for making scaffolds, was produced by melting in an electrically heated furnace. The reagents (analytical-grade powders of SiO_2_, CaCO_3_, Na_2_CO_3_, and Ca_3_(PO_4_)_2_ all purchased from Sigma–Aldrich, St. Louis, MO, USA) were homogeneously mixed in a platinum crucible and heated to 1500 °C for 1 h in air (heating rate 5 °C/min). The melt was quenched in deionized water to obtain a frit that was ball milled (Pulverisette 0, Frtisch, Germany) and sieved by a stainless-steel sieve (Giuliani Technology Srl, Italy) to obtain a final particle size below 32 μm. According to previous studies, this particle size range is very suitable to produce porous glass-derived scaffolds by sponge replication [26]. 

Disc-shaped scaffolds were then fabricated using an open-cell polyurethane foam (45 ppi; apparent density 18 kg/m^3^) as a sacrificial template. The basics and processing schedule of this fabrication method were described in detail elsewhere [19]. The sponge, which was previously cut in the form of discs (dimeter 30 mm, thickness 10 mm) was dipped into an aqueous suspension of 45S5 glass powder. Poly(vinyl alcohol) (PVA), used as a binder, was dissolved in deionized water at 80 °C under magnetic stirring for 30 min prior to the addition of glass powder; the water evaporated during PVA dissolution was re-added to keep constant the solid-to-liquid ratio for each sample during the manufacturing process. As the aim of this work was to study the relationships between elastic properties and porosity of the scaffolds, samples with different porosities were produced by varying the weight ratio of the slurry components (glass: PVA: water = x: 6: (100 – (x + 6))). Specifically, the solid load x was increased from 27 to 45 wt.% with a step of 2 wt.%; as a result, ten samples with different total porosity were obtained and denoted by the code “S-x”. After being extracted from the slurry, the glass-coated sponge was squeezed twice by applying an homogenous pressure (14.1 kPa, generated by a mass of 1.0 kg) on its circular faces in order to remove the excess suspension from the pores and leave a thin layer of glass particles on the template struts. This sequence of dipping–squeezing cycles was repeated thrice. The samples were left to dry overnight at room temperature in air and all were thermally treated under the same condition (1180 °C for 3 h) to remove the sacrificial polymer and sinter the glass particles.

### 2.2. Characterizations

The sintered material was ground in powder and analyzed by X-ray diffraction (XRD) in the 2θ-range of 10 to 60°; for this purpose, a X’Pert Pro PW3040/60 diffractometer (PANalytical. Eindhoven, The Netherlands) was used (operating parameters: voltage 40 kV, current 30 mA, Cu Kα incident radiation (λ = 0.15405 nm), step size 0.02°, fixed counting time 1 s per step). Crystalline phase assignment was performed using X’Pert HighScore software (2.2b) equipped with the PCPDFWIN database.

The scaffold morphology was investigated by scanning electron microscopy (SEM). After being sputter-coated with chromium, the sample was analyzed by field-emission SEM (Supra^TM^ 40, Zeiss, Oberkochen, Germany) at an accelerating voltage of 15 kV.

The total fractional porosity *p* of each scaffold was determined by mass-volume experimental measurements as follows:

(1) $$p=1-\frac{\rho }{\rho_{0}}\;$$

where ρ is the density of the scaffold and ρ_0_ is the density of the bulk (non-porous) material. The density was calculated as the mass-to-volume ratio of the scaffold (sample geometry was measured using digital calipers).

The elastic modulus (*E*), shear modulus (*G*), and Poisson’s ratio (*ν*) of the scaffolds were non-destructively determined by making use of the GrindoSonic system (MK5, Leuven, Belgium), which implements the impulse excitation technique. This test method is useful to determine the dynamic elastic properties of ceramics at ambient temperature. Specimens of these materials possess specific mechanical resonant frequencies that depend on the elastic properties, mass, and geometry of the test specimen. The GrindoSonic equipment measures the fundamental resonant frequency of test specimens by exciting them mechanically by a single elastic strike with an impulse tool; the experimental setup and method details are illustrated in the relevant ASTM standard [27].

The use of disc-shaped samples with a dimeter-to-thickness ratio of 3: 1 is recommended for the assessment of these elastic properties; this is the reason why scaffolds with this geometry were fabricated in this work, as described in Section 2.1. The discs were manually polished using SiC #1000 grit paper prior to being tested. 

The compressive strength was evaluated by crushing tests on the polished porous discs (MTS testing machine, MTS Corporation, Eden Prairie, MN, USA; 5 kN cell load, cross-head speed 1 mm/min) as:

(2) $$\sigma =\frac{L}{A}$$

where *L* (N) was the maximum load registered during the test and *A* (mm^2^) was the resistant cross-sectional area measured prior to the test by using digital calipers. 

## 3. Results and Discussion

**Figure 1:**
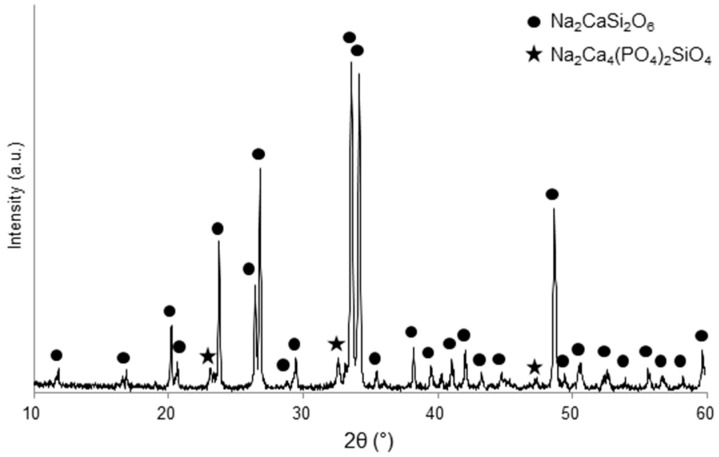
XRD pattern of 45S5-derived glass–ceramic scaffolds.

The XRD pattern shown in Figure 1 provides evidence of the glass–ceramic nature of the scaffolds, thus revealing that 45S5 glass underwent devitrification during sintering. This is consistent with previous literature, as the high tendency of 45S5 to crystallize above 550 °C has been demonstrated in several studies [28,29,30]. The main crystalline phase In Figure 1 was identified as combeite (Na_2_CaSi_2_O_6_, PDF code 01-077-2189), and silico-rhenanite (Na_2_Ca_4_(PO_4_)_2_SiO_4_, PDF code 00-032-1053) was also detected as a secondary phase. These results are in good accordance with previous studies about the sinter-crystallization of 45S5 Bioglass^®^ powder [26,31,32]. 

**Figure 2:**
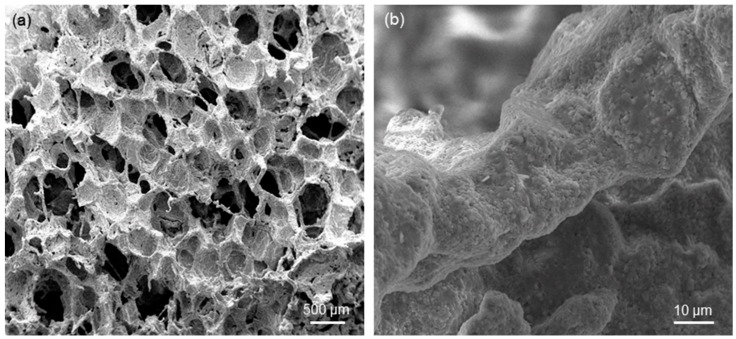
SEM micrographs showing the morphology of 45S5-derived foam-like scaffolds (S-41 sample): (**a**) overview of pore-strut architecture (magnification 50×) and (**b**) detail of a sintered strut (magnification 3000×).

A successful replica of the polymeric template (foam) was obtained after sintering, as shown in Figure 2a. There is abundant literature demonstrating that sponge replication is a highly effective and relatively simple method to produce ceramic and glass scaffolds with a trabecular architecture mimicking that of cancellous bone. Figure 2b shows that scaffold struts are well densified and exhibit a diffused surface micro-roughness, which can be useful to promote osteoblastic cell attachment in bone tissue engineering applications [33]. 

The total porosity and elastic properties of the samples are collected in Table 1. As expected, higher the solid load of the slurry used for scaffold preparation, lower the total porosity.

The values of elastic modulus were found to decrease as the scaffold porosity increased and, in general, were comparable or superior to the typical range of human cancellous bone. In this regard, the elastic modulus of bone varies greatly depending on the patient’s harvesting site, sex, and age (0.05–0.5 GPa in Reference [34] and 0.5–5.0 GPa in Reference [35]). 

The relationship between elastic modulus and total porosity in the scaffolds can be described through the power-law model developed by Gibson and Ashby [36]. They demonstrated that the elastic modulus of low-density cellular solids is controlled by the relative density as follows:

(3) $$\frac{E}{E_{0}}=C{\left(\frac{\rho }{\rho_{0}}\right)}^{n}=C{\left(1-p\right)}^{n}$$

where *E*_0_ is the elastic modulus of the non-porous solid, and *C* and *n* are constants that depend on the microstructure. For cellular structures with a dense solid network, we have *C* = 1, while in other cases, this constant can assume different values (for example *C* = 0.3 for ceramic network with a central empty channel) [36]. The exponent *n* has a value in the range of 1 to 4, with *n* = 2 for open-cell structures. The 45S5-based scaffolds can be approximated as open-cell structures in which the solid network was fully dense; therefore, we have *C* = 1 and *n* = 2 and Equation (3) can be rewritten as:

(4) $$E=E_{0}{\left(1-p\right)}^{2}$$

**Figure 3:**
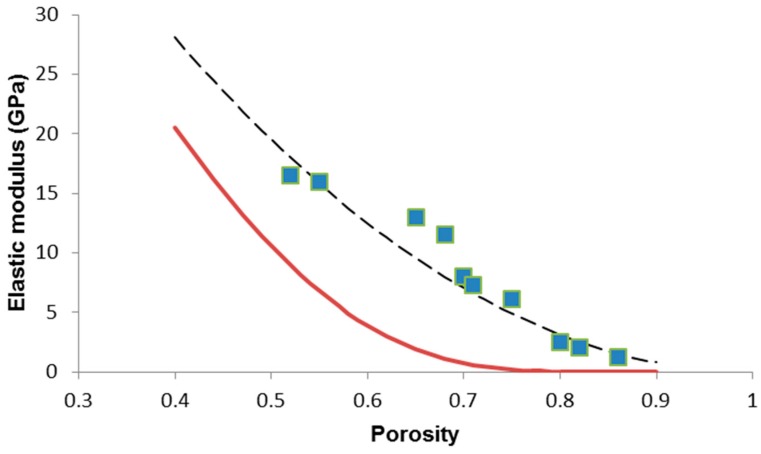
Porosity dependence of elastic modulus in 45S5 glass-derived foams with data interpolation by applying the Gibson–Ashby power-law model (Equation (4)) (dashed line) and the Pabst–Gregorova exponential relation (Equation (6)) (solid line).

The elastic modulus of the bulk glass (*E*_0_ = 78 GPa) was estimated by means of the software SciGlass (AKos, Steinen, Germany) implementing the analytical method Priven 2000. The result of the data fitting is reported in Figure 3; the high value of the correlation coefficient (*R*^2^ = 0.8953), calculated by implementing the least squares method in MATLAB (MathWorks, Natick, MA, USA), suggests the good predictive capability of the power-law model.

Other analytical relations have been proposed to describe the porosity dependence of elastic modulus in porous ceramics; for example, Pabst and Gregorova [37] developed the following simple and very elegant equation:

(5) $$E=E_{0}\left(1-2p\right)$$

However, this linear approximation is valid only for ceramics with low pore content and, thus, cannot be applied to highly porous scaffolds with *p* > 0.50.

The same authors also proposed a more general exponential relation [37]:

(6) $$E=E_{0}\exp\left(\frac{-2p}{1-p}\right)$$

The validity of which has been confirmed, for example, in experimental studies on ceramics produced by starch consolidation [38] and alumina/zirconia composites [39].

The result of the fitting of experimental data through Equation (6) is illustrated in Figure 3: apparently, the Pabst–Gregorova exponential model seems unsuitable to describe the relationship between elastic modulus and porosity in 45S5 glass-derived foams, as also confirmed by the correlation coefficient (*R*^2^ = 0). 

**Figure 4:**
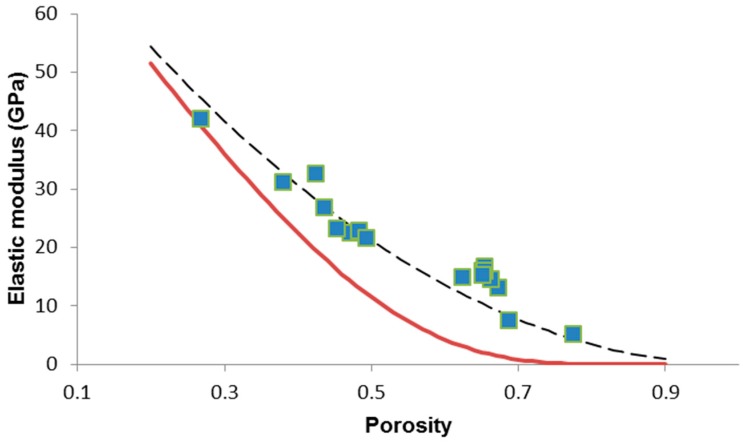
Porosity dependence of elastic modulus in CEL2 glass-derived foams with data interpolation by applying the Gibson–Ashby power-law model (Equation (4)) (dashed line) and the Pabst–Gregorova exponential relation (Equation (6)) (solid line). Raw data from Reference [41].

We tried applying the same models (Equations (4) and (6)) to other sets of data on bioactive glass scaffolds for the purpose of comparison. A review of existing literature shows that there is a paucity of elastic data on large numbers of bioactive glass scaffolds [40]; however, we found that Malasoma et al. [41] reported the porosity and elastic modulus of several SiO_2_–Na_2_O–CaO–MgO–K_2_O–P_2_O_5_ glass (CEL2) scaffolds produced by the sponge replica method. The results of the data fitting are shown in Figure 4 and, also in this case, the power-law model with *n* = 2 seems very suitable to describe the relationship between elastic modulus and porosity (*R*^2^ = 0.8378), unlike the Pabst–Gregorova approach (*R*^2^ = 0). 

This finding can be explained considering that the Pabst–Gregorova exponential model was developed assuming the presence of perfectly spherical pores, which is not the case of foam-like structures derived from an open-cell polymeric template. The Pabst–Gregorova approach could perhaps be more suitable to describe the relationship between elastic modulus and porosity of bioactive glass scaffolds produced by sol-gel foaming [42,43,44], as this fabrication method actually introduces spherical macropores deriving from entrapped air bubbles in the material; this would deserve to be verified in a future study. 

The data reported in Table 1 suggest an increasing trend of the Poisson’s ratio as the porosity increases. The Poisson’s ratio can be expressed according to the following equation [45]:

(7) $$\nu =0.5-\frac{E}{6K}$$

where *K* depends on material porosity.

For highly porous ceramics (*p* > 0.40), *K* is a function of *p*, the bulk modulus (*K*_0_) and the Poisson’s ratio of the pore-free material (ν_0_) as follows [45]:

(8) $$K=K_{0}\frac{2\left(1-2\nu_{0}\right)\left(1-p\right)}{3\left(1-\nu_{0}\right)}$$

where $K_{0}=\frac{E_{0}}{3\left(1-2\nu_{0}\right)}$.

Thus, after substituting the Equations (4) and (8) in Equation (7), we obtain:

(9) $$\nu =0.5-\frac{3}{4}\left(1-\nu_{0}\right)\left(1-p\right)$$

**Figure 5:**
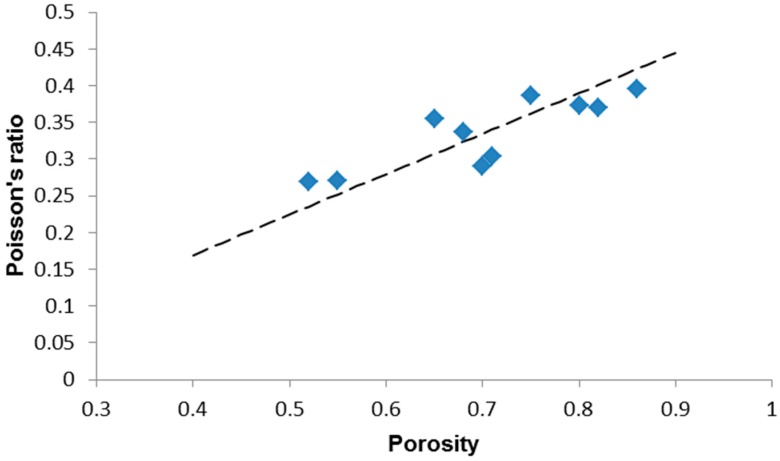
Porosity dependence of the Poisson’s ratio in highly porous 45S5 glass-derived foams with data interpolation by a linear model (Equation (9)).

The Poisson’s ratio of the bulk material (*ν*_0_ = 0.2651) was estimated by means of the software SciGlass implementing the analytical method Priven 2000. The result of the data fitting is reported in Figure 5: a linear relationship can be suggested, also considering the moderately high value of the correlation coefficient (*R*^2^ = 0.5489). This trend is in agreement with the results reported by Arnold et al. [45] for highly porous gel-derived silica. It is interesting to underline that the porosity dependence of Poisson’s ratio varies according to the level of porosity considered. In fact, for *p* < 0.40, the Poisson’s ration tends to decrease as porosity increases, as shown by De With et al. [46] who prepared and analyzed porous hydroxyapatite with porosity ranging from 0.03 to 0.27: this is an opposite trend compared to that exhibited by highly porous ceramics which still needs to be fully elucidated.

According to Gibson and Ashby [47], the porosity dependence of shear modulus in open-cell ceramic foams is analogous to that of the elastic modulus:

(10) $$G=\gamma G_{0}{\left(1-p\right)}^{2}$$

where *G*_0_ is the shear modulus of the non-porous solid and $\gamma =\frac{3\left(1+\nu_{0}\right)}{4}$.

**Figure 6:**
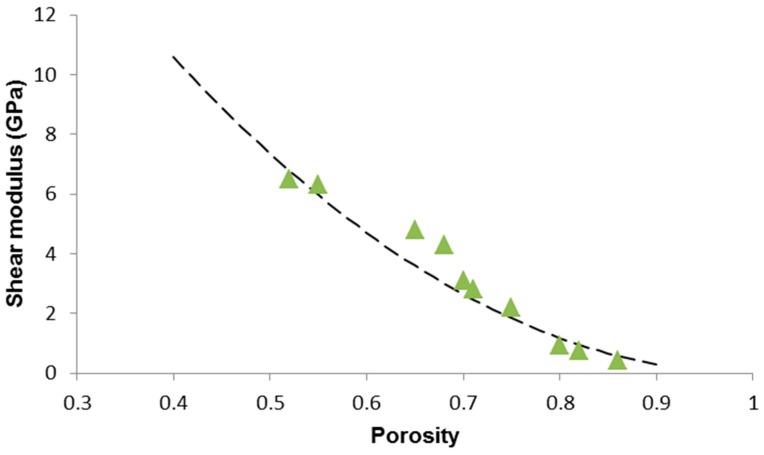
Porosity dependence of shear modulus in 45S5 glass-derived foams with data interpolation by applying the Gibson–Ashby power-law model (Equation (10)).

The shear modulus of the bulk material (*G*_0_ = 31 GPa) was estimated by means of the software SciGlass. The result of the data fitting is reported in Figure 6: the high value of the correlation coefficient (*R*^2^ = 0.8491) suggests the suitability of the power-law model to describe the experimental data.

The compressive stress–strain curves of the scaffolds exhibited the typical profile of cellular ceramics, having a multi-peak trend related to multiple fracture events which occur during the crushing test [10,19,42]. The maximum peak corresponded to a strain below 10% for all the specimens; this is in line with the observations reported by Denry et al. [48] who pointed out that, due to the typical step-wise failure mode of macroporous scaffolds, only the peaks corresponding to a displacement equal or lower than 30% of the height of the specimen may be appropriate for the determination of the breaking strength. 

As shown in Table 1, 45S5 scaffolds with porosity above 0.60 have a compressive strength below the typical standard range assumed for the human trabecular bone (2–12 MPa [11]). However, the lowest value of strength (0.52 MPa for *p* = 0.86) is two times higher than that reported for analogous 45S5 Bioglass^®^ foams produced by sponge replication and sintered at 1100 °C for 1 h [10]. This can be explained considering that the higher temperature (1180 versus 1100 °C) and longer time (3 versus 1 h) used for the sintering process allowed obtaining a better densification of the struts; these results are consistent with those assessed on the same porous material in a previous work [49].

The power-law approach is often used to describe the mechanical response of cellular foams [36]:

(11) $$\sigma =\sigma_{0}C_{1}{\left(1-p\right)}^{m}$$

where σ_0_ is the compressive strength of the bulk material, and *C_1_* and *m* are the model constant.

**Figure 7:**
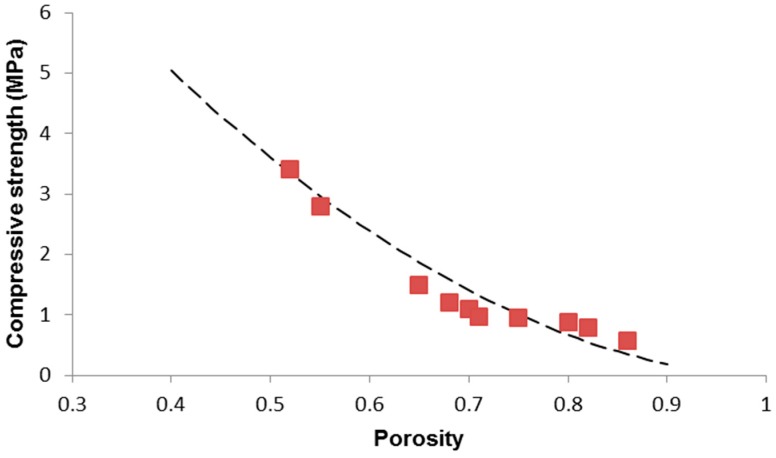
Porosity dependence of compressive strength in 45S5 glass-derived foams with data interpolation by applying the Gibson–Ashby power-law model (Equation (11)).

The interpolation of the experimental data to estimate *C_1_* and *m* was carried out using a proper code developed in MATLAB and based on the least squares method. The results of model fitting, carried out assuming σ_0_ = 500 MPa [50], is reported in Figure 7. The fitted parameters are *C_1_* = 0.0258 and *m* = 1.84; the coefficient *R*^2^ = 0.9165 suggests a good accuracy and predictive capability of the power-law model for the compressive strength, too. The exponent *m* is also comparable to that assessed for human trabecular bone by other authors [51].

## 4. Conclusions

Glass–ceramic scaffolds based on the 45S5 bioactive glass composition were fabricated by the sponge replica method. These porous materials could be considered potentially suitable for bone substitution from a mechanical viewpoint as their elastic modulus and compressive strength were comparable to those of human trabecular bone. The relationships between scaffold porosity and elastic modulus, shear modulus, Poisson’s ration, and compressive strength were investigated and modelled through different approaches. Data fitting suggests that the porosity dependences of elastic modulus, shear modulus and compressive strength obeys power-law relations, whereas an increasing linear trend could describe the relationship between Poisson’s ratio and porosity. A limitation of this study is the relatively low number of samples tested experimentally; however, the high values of *R*^2^ coefficients show promise and support these results, which deserve to be confirmed in future studies on a larger scaffold number. In principle, these relations are valuable for the development of mechanically optimized scaffolds: knowing the porosity, which is relatively easy to determine, they can be used to predict the scaffold mechanical properties; alternatively, using the mechanical property as a model input, the scaffold manufacturer can estimate the porosity which is required to achieve that mechanical performance. Hence, the fabrication process can be properly optimized to obtain that mechanical property, thereby saving manufacturing time and cost.

## Figures and Tables

**Table 1:** Porosity and mechanical properties of the 45S5-based scaffolds.

Scaffold Code (S-x)	*p*	*E* (GPa)	*G* (GPa)	*ν*	*σ_c_* (MPa)
S-27	0.86	1.2	0.43	0.3953	0.58
S-29	0.82	2.0	0.73	0.3698	0.78
S-31	0.80	2.5	0.91	0.3736	0.88
S-33	0.75	6.1	2.2	0.3863	0.95
S-35	0.71	7.3	2.8	0.3035	0.96
S-37	0.70	8.0	3.1	0.2903	1.1
S-39	0.68	11.5	4.3	0.3372	1.2
S-41	0.65	13.0	4.8	0.3541	1.5
S-43	0.55	16.0	6.3	0.2698	2.8
S-45	0.52	16.5	6.5	02692	3.4
